# Suppression of Light-Induced Retinal Degeneration by Quercetin via the AP-1 Pathway in Rats

**DOI:** 10.3390/antiox8040079

**Published:** 2019-03-27

**Authors:** Yasurou Koyama, Sachiko Kaidzu, Yong-Chul Kim, Yotaro Matsuoka, Tomoe Ishihara, Akihiro Ohira, Masaki Tanito

**Affiliations:** 1 Department of Ophthalmology, Faculty of Medicine, Shimane University, Shimane 693-0021, Japan; ykoyama@med.shimane-u.ac.jp (Y.K.); ymatsu@med.shimane-u.ac.jp (Y.M.); aohira@med.shimane-u.ac.jp (A.O.); mtanito@med.shimane-u.ac.jp (M.T.); 2 Department of Pathology, Uniformed Services University of the Health Science, Bethesda, MD 20814, USA; yongchul.kim.ctr@usuhs.edu; 3 Department of Research and Development, Kotobuki Seika Co., Ltd., Tottori 683-0845, Japan; t-ishihara@kozuchi-net.jp

**Keywords:** quercetin, light damage, oxidative stress, retina, AP-1

## Abstract

We examined the cytoprotective effect of quercetin via activator protein (AP-1) and the heat shock protein 70 (Hsp70) pathway against light-induced retinal degeneration in rats. Quercetin was administered intraperitoneally to Sprague-Dawley rats for seven days before light exposure to intense white fluorescent light (3000 lux) for 24 h. Light-induced retinal damage was determined by the number of rows of photoreceptor cell nuclei, the microstructures of the rod outer segments and retinal pigment epithelium, and terminal deoxynucleotidyl transferase (TdT)-mediated 2′-Deoxyuridine-5′-triphosphate (dUTP) nick end labeling. To elucidate the cytoprotective mechanism of quercetin, expression levels were measured in the rat retinas of 8-hydroxy-deoxyguanosine (8-OHdG), a marker of oxidative stress; Hsp70; and transcription factor AP-1 transcription activity. Pretreatment with quercetin inhibited light-induced photoreceptor cellular apoptosis and subsequent retinal degeneration in rats. 8-OHdG and Hsp70 protein expressions were up-regulated markedly by light exposure and suppressed by quercetin pretreatment. The results of an electrophoretic mobility shift assay showed that AP-1-binding activity was activated by light exposure, and binding of c-Fos and c-Jun, but not JunB, mediated the binding activity. Intraperitoneal administration of quercetin decreases photooxidative damage in the retina and mediates cytoprotection against light-induced photoreceptor cell degeneration in rats. Suppression of the heterodimeric combination of c-Jun and c-Fos proteins at the AP-1 binding site is highly involved in quercetin-mediated cytoprotection.

## 1. Introduction

Exposure to excessive light induces apoptotic cellular death of the photoreceptors in albino rats [1,2] and mice [3,4], and reactive oxygen species are important factors in this light-induced apoptosis [5,6]. The c-Fos component of the transcription factor activator protein (AP-1) is involved in the light-induced photoreceptor apoptosis. Retinal expression of c-Fos is up-regulated transiently by light exposure in rats [3,7] and mice [8]; photoreceptors of c-Fos-deficient mice (c-fos−/−) are highly resistant to light-induced damage by blocking apoptosis, indicating that c-Fos is essential for light-induced apoptosis of photoreceptors [9,10].

Quercetin is a plant polyphenolic compound found widely in various fruits and vegetables. Quercetin and its metabolites are potent antioxidants [11,12], with oxygen radical scavenging properties [13], and inhibit lipid peroxidation in vitro [14]. In ocular tissues, quercetin and its metabolites also protect against oxidative stress in the rat lens [15] and cultured chick retinal cells [16], human retinal pigment epithelium (RPE) cells [17,18], and bovine retina [19], and prevent lipid peroxidation in bovine and porcine rod outer segments (ROS) [20].

Quercetin also affects several signal transduction pathways and/or endogenous anti-stress molecules, such as suppression of both the expression of heat shock-mediated heat shock protein 70 (Hsp70) and c-Fos in astrocytes [21] and the extracellular signal-regulated kinase c-Fos/AP-1 pathway of apoptosis in renal mesangial cells [22]. While the precise pathway is unclear in the ischemia-reperfusion injury rat model, quercetin actually reduces apoptosis in the retina [23]. Therefore, quercetin also is expected to reduce light-induced apoptosis via AP-1. However, no previous report has investigated whether quercetin suppresses light-induced apoptosis in vivo.

Retinal tissue is affected easily by oxidative stress because it is comprised of a unique fatty acid component and has the highest oxygen consumption [24]. Oxidative stress is involved in age-related macular degeneration (AMD), which is the primary risk factor for visual loss with the progression of aging [25]. However, there is no effective medical treatment for AMD and preventive medicine is required. It is reported that dietary treatment with antioxidants delays disease progression [26]. As quercetin has various functions, it was expected to become the candidate of preventive medicine with multifunction. The current study is the first to test the cytoprotective effect of quercetin via AP-1 and the Hsp70 pathway against light-induced retinal degeneration model in rats. In this model, oxidative stress induced retinal damage. To elucidate the cytoprotective mechanism of quercetin, we measured the expression levels of 8-hydroxy-deoxyguanosine (8-OHdG), a marker of oxidative stress-induced cellular damage, Hsp70 protein, and AP-1 transcription activity in rat retinas.

## 2. Materials and Methods

### 2.1. Animals

All procedures adhered to the Association for Research in Vision and Ophthalmology (ARVO) Statement for the Use of Animals in Ophthalmic and Vision Research; the Animal Care and Use Committee in Shimane University approved all protocols (IZ26-188). Sprague-Dawley rats (6 weeks old) were obtained from Charles River Japan, Inc. (Kanagawa, Japan) and maintained in our colony room on a cyclic light cycle (12 h on/off, 7 a.m.–7 p.m.) at 22 to 24 °C for 2 weeks before the experiments. The illumination intensity during the light phase was 80 lux. Water and Purina Rat Chow (Nestle SA, Vevey, Switzerland) were provided ad libitum. One animal was housed in each cage, which had wire tops and minimal bedding.

### 2.2. Quercetin Administration

The rats were divided into the quercetin-treated (+) (316.9 ± 7.4 g body weight) and untreated (−) groups (315.3 ± 7.4 g body weight) (*n* = 60 in each group). In the quercetin (+) group, quercetin (Sigma, St. Louis, MO, USA) was suspended in physiologic saline (50 mg/mL) and administered intraperitoneally (50 mg/body weight/kg) once daily for 6 days and 2 h before light exposure. The dosage was determined based on a previous report [27]. In the quercetin (−) group, physiologic saline was administered intraperitoneally (1 mL/ body weight/kg) once daily for 6 days and 2 h before light exposure. We used 6 rats for electroretinography (ERG) and histology (hematoxylin & eosin [HE] and immunostaining) and 4 rats for electron microscopy (right eyes), Western blotting, and an electrophoretic mobility shift assay (EMSA) (left eyes) at each time point (*n* = 10).

### 2.3. Light Exposure Model

The light exposure experiment was performed based on the methods of Yamamoto and associates with minor modification [28]. Briefly, 8-week-old Sprague-Dawley rats were exposed to white fluorescent light; the average light intensity at 10 randomly selected points in the cages was 3000 lux for 24 h. All experiments began at 9 a.m. because the ROS phagocytosis process by RPE cells is controlled by circadian rhythm, and this activity peaks 2 h after the start of the light cycle [29,30]. The pupils were dilated with 0.5% tropicamide and 0.5% phenylephrine hydrochloride eye drops (Santen Pharmaceuticals Co., Ltd., Osaka, Japan). The temperature during the illumination was maintained at 25 ± 1.5 °C. After light exposure, the animals were maintained in cyclic light (12 h on/off, 7 a.m.–7 p.m.) until subjected to ERG and enucleation. Retinal samples were collected 12 and 24 h after light exposure started and 1, 3, and 7 days after exposure finished.

### 2.4. ERGs

Seven days after the light exposure ended, flash ERGs were recorded (LS-W, Mayo Corporation, Aichi, Japan). Twenty minutes before the recording, anesthesia was induced by intraperitoneal injection of a mixture of ketamine (120 mg/kg) and xylazine (6 mg/kg), and the pupils were dilated by the same method described previously. Light-emitting diode (LED) electrodes (Mayo Corporation), which were connected to contact lens electrodes, were placed on both eyes to lightly touch the cornea. An identical reference electrode was placed in the mouth, and the ground electrode was placed on the left footpad. A single flash of light (10,000 cd/mm^2^, 5 ms) from the LED was used as light stimulation. The a and b-wave amplitudes from both eyes were measured, and the average values were used for statistical analysis.

### 2.5. Preparation of Retinal Paraffin Sections

For histologic and immunohistologic analyses, the anesthetized animals were perfused with ice-cold phosphate buffer (0.1 M; pH 7.4) through the left cardiac ventricle and then perfused with freshly prepared 2% paraformaldehyde, 0.1% glutaraldehyde, and 1% sucrose in the same buffer. The left eyes then were removed and fixed with the same fixative for 6 h at 4 °C. The right eyes were used for electron microscopy analysis. All tissues were embedded in paraffin wax and cut into 4-μm-thick sections that were parallel to the sagittal line and included the optic nerve head (ONH).

### 2.6. Light-Induced Retinal Damage

To analyze the severity of retinal damage, the numbers of rows of nuclei in the outer nuclear layers (ONL) and the ONL thickness were measured on the retinal sagittal sections stained with HE, as previously reported [31].

### 2.7. Terminal Deoxynucleotidyl Transferase (TdT)-Mediated 2′-Deoxyuridine-5′-Triphosphate (dUTP) Nick End Labeling (TUNEL)

TUNEL staining was performed on the retinal sections using an apoptosis in situ detection kit (Wako Pure Chemicals, Osaka, Japan) with some modification of the manufacturer’s protocol. Briefly, deparaffinized sections were washed with distilled water and treated with Protein Digestion Enzyme (Wako Pure Chemicals, Osaka, Japan) for 10 min at 37 °C. After washing with phosphate buffered saline (PBS), the sections were treated with TdT solution, incubated with 3% hydrogen peroxide for 5 min at room temperature to block endogenous peroxidase activity, and immersed in blocking solution (3% casein, and 1% bovine serum albumin in PBS containing 0.05% Tween 20^®^, AMRESCO, Ohio, USA) for 60 min at room temperature. The sections were treated with peroxidase conjugated antibody for 10 min at 37 °C. After washing with PBS, nick end labeling was visualized by immersing reacted sections in 0.05% diaminobenzidine solution with 0.01% hydrogen peroxide. As a negative control, the tissue sections were incubated with TdT buffer that did not contain the enzyme. For the positive control, tissue sections were treated with Deoxyribonuclease I (DNase I) before treatment with TdT.

### 2.8. Immunohistochemistry for 8-OHdG

Expression of 8-OHdG, an established marker of oxidative stress-induced DNA damage [32,33], was determined by immunohistochemistry performed on the retinal sections. Deparaffinized sections were subjected to microwaving (NE-N2, National, Tokyo, Japan) in 10 mM citrate buffer (pH 6.0) for 10 min and treated with 3% H_2_O_2_ for 10 min. After washing with PBS, the sections were immersed in the blocking solution (10% normal rabbit serum) for 10 min at room temperature. The sections were incubated with primary antibodies (anti-8-OHdG antibody; N45.1, NOF Corporation, Tokyo, Japan) or normal mouse serum (1/200) overnight at 4 °C, followed by incubation with biotinylated rabbit Immunoglobulin G (IgG) against mouse IgG for 10 min at room temperature. The sections then were reacted with avidin-biotinylated peroxidase complex (Histofine, Nichirei, Tokyo, Japan) for 5 min at room temperature. Specific labeling for 8-OHdG was visualized with 0.05% 3-3′ diaminobenzidine tetrahydrochloride (Dojindo, Kumamoto, Japan) and 0.01% H_2_O_2_ in 0.05 M Tris-HCl buffer (pH 7.6) for 5 min.

### 2.9. Electron Microscopy

The right eyes were enucleated, fixed with the same fixative described previously, and washed in 0.1 M phosphate buffer (pH 7.4). Retinal pieces were cut parallel to the sagittal line and included the ONH, and then pieces were cut into 4 parts (total 8 parts). The 2 most superior pieces (superonasal and superotemporal) were further cut with a diameter of about 2 mm including the ONH (the peripheral retina was cut off), postfixed in 2% osmium tetroxide for 2 h, and dehydrated in a graded series of ethanol. The pieces were embedded in epoxy resin and trimmed until the retina, with the exception of about 450 μm from the ONH, was seen. We selected the better piece (superonasal or superotemporal) without a retinal detachment, based on observation of semi-thin sections (~900 nm) stained with toluidine blue. Ultra-thin sections (~90 nm) were cut from the retina every 50 μm (450, 500, and 550 μm from the ONH), post-stained with uranyl acetate and lead citrate, and examined and photographed by transmission electron microscopy (TEM) (TEM-002B, Topcon, Japan). We observed 4 retinas from 4 rats, obtained 3 photos from 1 retina, and counted the number of phagosomes in a 20-µm baseline of RPE. A phagosome was defined as the presence of lamellar structures in the RPE surrounded by microvilli and lysosomes (>1 µm) (*n* = 12 in each group). 

### 2.10. Western Blotting for Hsp70

The methods of retinal and RPE sample preparation and Western blotting have been described previously [34]. Briefly, after deep anesthesia was induced by pentobarbital, the rats were perfused through the left cardiac ventricle with ice-cold 0.1 M phosphate buffer (pH 7.4) to wash out the blood, and the eyes were removed. After the cornea and lens were removed, the neural retinas were separated from the eyecups under microscopic view. The eyecups were analyzed as a RPE fraction, which also contained the choroids and sclera. Equal amounts of protein (20 µg protein/lane) were electrophoresed on 10% sodium dodecyl sulfate-polyacrylamide gel and then electrophoretically transferred to a polyvinylidene difluoride membrane (Millipore, Bedford, MA). After the membrane was blocked, it was incubated with the first antibodies (mouse anti-Hsp70, StressGen Biotechnologies Inc., Victoria, British Columbia, Canada) and then with the peroxidase-linked second antibody. Chemiluminescence was detected with an Enhanced Chemiluminescence (ECL) Western blot detection kit (Amersham Pharmacia Biotech, Buckinghamshire, UK). To confirm that equal amounts of protein were electrophoresed and transferred to the membrane, staining with Coomassie Brilliant Blue (CBB) R-250 was performed. We used 4 rats in each group. The left eyes were used for Western blotting and the right eyes were used for the EMSA.

### 2.11. EMSA

EMSA was performed as described previously [30,31]. Rabbit anti-c-Fos (K-25), -c-Jun (D), and -JunB antibodies were purchased from Santa Cruz Biotechnology (Santa Cruz, CA). Anti-c-Fos antibody recognizes c-Fos, FosB, Fos related antigen (Fra) -1, and Fra-2. Anti-c-Jun antibody recognizes c-Jun, JunB, and Jun D. Nuclear protein was extracted from the neural retinas using a Nuclear/Cytosol Fractionation Kit (BioVision, Mountain View, CA). Aliquots of 10 µg of nuclear extract were incubated with 32P-end-labeled double-stranded AP-1 oligonucleotides (E3201, Promega, Madison, WI, USA) in a binding reaction buffer at 25 °C for 20 min. For specificity analyses, 100-fold molar excess of unlabeled oligonucleotide competitors was added and preincubated for 15 min. When indicated, the reaction mixtures were incubated with antibodies for 20 min on ice before labeled oligonucleotides were added.

### 2.12. Statistical Analysis

All data are expressed as the means ± standard deviations (SDs). Comparisons between quercetin (+) and (−) groups were performed using the Mann-Whitney U-test. Multiple group comparisons in the time-course analyses were performed by one-way analysis of variance (ANOVA) with StatMate software (ATMS Co., Tokyo, Japan) followed by the Tukey test. *p* < 0.05 was considered significant.

## 3. Results

### 3.1. ERGs

**Figure 1:**
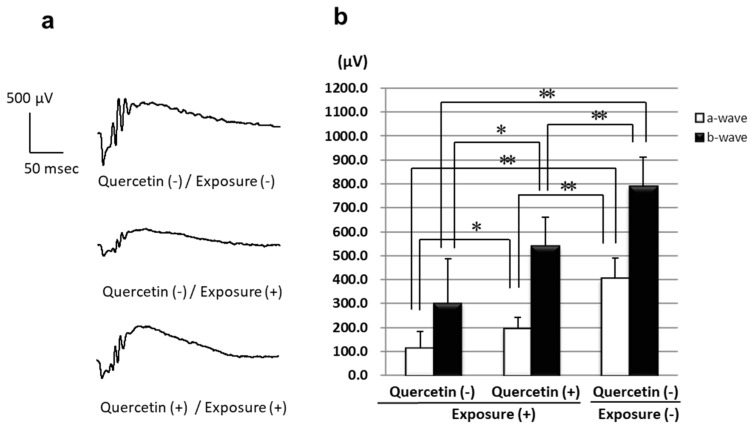
(**a**) Representative electroretinography (ERG) recordings and (**b**) a- and b-wave amplitudes are shown. The data are expressed as the mean ± standard deviation (SD) (*n* = 6 in each group). While a and b-wave amplitudes decreased significantly as the result of light exposure, the amplitudes were suppressed partly in the quercetin (+) group. * *p* < 0.05, ** *p* < 0.01 by the Turkey test.

ERGs were recorded 7 days after light exposure to estimate the retinal function. In the quercetin (+)/exposure (−) group, the a and b-wave amplitudes were significantly higher than those in the quercetin (−)/exposure (+) group, although lower than that in the quercetin (−)/exposure (−) group (Figure 1).

### 3.2. Photoreceptor Degeneration with Light Exposure

**Figure 2:**
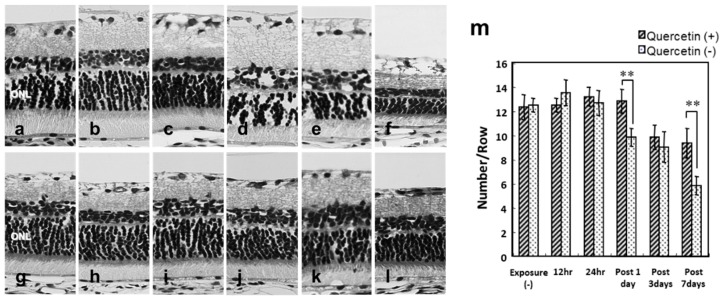
Light-induced retinal damage. Superior retinal samples obtained 500 μm from the optic nerve head (ONH) are seen. (**a**–**f**) In the quercetin (−) retinas, the retinal thickness and photoreceptor cell nuclei in the outer nuclear layer (ONL) are decreased after light exposure (**a**–**f**,**m**). (**g**–**l**,**m**) In quercetin (+) retina, the decreases in thickness and nuclei are suppressed partly. (**a**,**g**) Unexposed retinas; (**b**,**h**) the retinas were exposed to light for 12 h; (**c**,**i**) the retinas exposed to light for 24 h; (**d**,**j**) 1 day after light exposure; (**e**,**k**) 3 days after light exposure; (**f**,**l**) 7 days after light exposure. Hemetoxylin & Eosin (HE) staining. Bar = 25 μm. The data are expressed as the mean ± SD, *n* = 6 in each group. ** *p* < 0.01 by the Mann-Whitney U test. hr, hours.

In the quercetin (−) group, the number of photoreceptor cell nuclei was significantly lower 1 day after light exposure and compared to the retinas not exposed to light (Figure 2d,m). Seven days after light exposure, the mean number of photoreceptor cell nuclei was 5.8 ± 0.8, which was almost half that of the normal retina (12.5 ± 0.6) (Figure 2m). The number of photoreceptor cell nuclei was significantly higher in the quercetin (+) group than in the quercetin (−) group 1, 3, and 7 days after light exposure (Figure 2d–f,j–l,m).

**Figure 3:**
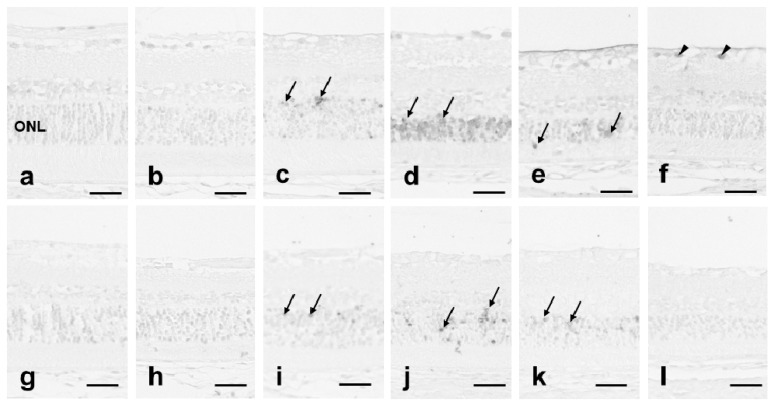
Terminal deoxynucleotidyl transferase (TdT)-mediated 2′-Deoxyuridine-5′-triphosphate dUTP nick end labeling. Superior retinal samples obtained 500 μm from the optic nerve are seen. (**a**–**f**) In quercetin (−) retina, TUNEL-positive cells are seen in the ONL (arrows) and ganglion cell layer (arrowheads). (**g**–**l**) In quercetin (+) retina, although some TUNEL-positive cells are seen in the ONL (arrows), there are much fewer than in the quercetin (−) retina. (**a**,**g**) Unexposed retinas; (**b**,**h**) retinas exposed to light for 12 h; (**c**,**i**) retinas exposed to light for 24 h; (**d**,**j**) 1 day after light exposure; (**e**,**k**) 3 days after light exposure; and (**f**,**l**) 7 days after light exposure. Bar = 25 μm. *n* = 6 in each group.

In the quercetin (−) group, no TUNEL-positive (+) cells were seen in normal and light-exposed retina for 12 h (Figure 3a,b). TUNEL (+) cells appeared immediately after 24 h of light exposure (Figure 3c), reached a maximal number 1 day after light exposure (Figure 3d), and subsided 3 days after light exposure (Figure 3e) and thereafter. Seven days after light exposure, no TUNEL (+) cells were seen in the ONL, but some ganglion cells had a TUNEL (+) reaction (Figure 3f). In the quercetin (+) group, TUNEL (+) cells were not seen in unexposed and light-exposed retina for 12 h (Figure 3g,h). Some cells in the ONL had a TUNEL (+) reaction in retinas exposed to light for 24 h (Figure 3i). One day after light exposure, there were much fewer TUNEL (+) cells than in the quercetin (−) retina (Figure 3d,j). Three days after exposure, the TUNEL (+) cells in the ONL decreased (Figure 3k). Seven days after exposure, no TUNEL (+) cells were seen throughout the retinal layer (Figure 3l).

The results indicated that light-induced photoreceptor cell apoptosis and subsequent retinal degeneration were more severe in the quercetin (−) group than in the quercetin (+) group.

### 3.3. 8-OHdG Expression

**Figure 4:**
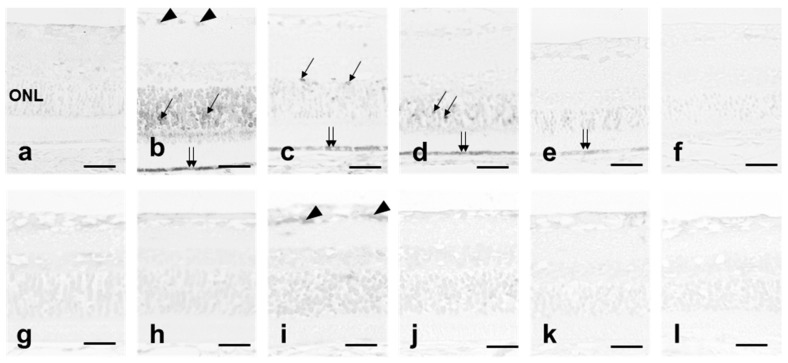
Expression of 8-hydroxy-deoxyguanosine (8-OHdG). Superior retinal samples obtained 500 μm from the optic nerve are seen. (**a**–**f**) In quercetin (−) retina, many cells in the ONL (arrows), ganglion cells (arrowheads), and retinal pigment epithelium (RPE) cells (double arrows) have evidence of oxidative stress resulting from light exposure. (**g**–**l**) The quercetin (+) retinas have mild oxidative stress. (**a**,**g**) Unexposed retinas; (**b**,**h**) retinas exposed to light for 12 h; (**c**,**i**) retinas exposed to light for 24 h; (**d**,**j**) 1 day after light exposure; (**e**,**k**) 3 days after light exposure; and (**f**,**l**) 7 days after light exposure. Bar = 25 μm. *n* = 6 in each group.

We evaluated 8-OHdG expression in the retinal sections to analyze the tissue level of photooxidative stress. In the quercetin (−) group, after 12 h of light exposure, the ganglion, photoreceptor, and RPE cells were labeled with 8-OHdG (Figure 4b). Most photoreceptor cells in the ONL were weakly labeled; however, some cells were strongly labeled. Immediately after 24 h of light exposure, labeling of those cells weakened or disappeared (Figure 4c). One day after light exposure, there was no change in the 8-OHdG labeling in the photoreceptor cells and RPE (Figure 4d). Three days after exposure, labeling in the photoreceptor cells almost disappeared, although some RPE cells were still labeled (Figure 4e) (arrows). Seven days after light exposure, labeling was not seen in the retinal cells, which appeared similar to normal retina (Figure 4a,f). However, in the quercetin (+) group, no labeling was seen in the retina during and after light exposure, with the exception of the ganglion cells immediately after 24 h of light exposure (Figure 4g–l). Some ganglion cells were slightly labeled immediately after light exposure (Figure 4i). No labeling was seen in the normal retinas in both groups (Figure 4a,g).

### 3.4. ROS Microstructure and Phagosomes in RPE

**Figure 5:**
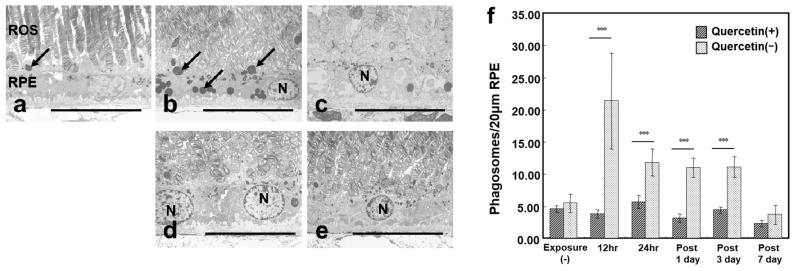
The rod outer segment (ROS) microstructure and phagosomes in the RPE. Superior retinal samples obtained from about 500 μm from the optic nerve are observed. (**a**) Unexposed RPE cells contain some phagosomes (arrow). (**b**) Quercetin (−) retinas have many phagosomes (arrows) after 12 h of exposure; (**c**) the arrangement of the ROS is disrupted 7 days after light exposure. (**d**) In quercetin (+) retina, the RPE cells are almost the same as the normal retinas during and (**e**) after exposure. (**f**) The number of phagosomes in 20-μm-long RPE has increased during and after exposure only in quercetin (−) retina. Bar = 5 μm. The data are expressed as the mean ± SD (*n* = 12). *** *p* < 0.001 by the Mann-Whitney U test. hr, hours; N: nucleus.

We evaluated the ultrastructural changes in the outer retina by electron microscopy to further confirm the cytoprotective effect of quercetin. In the normal retinas, some phagosomes were seen in the RPE cells (Figure 5a). After 12 h of light exposure, many phagosomes were seen in the RPE cells in the quercetin (−) retinas (Figure 5b,f); however, no increase in the number of phagosomes was seen in the quercetin (+) retinas (Figure 5d,f). Seven days after exposure, the arrangement of the ROS was disrupted markedly in the quercetin (−) retinas (Figure 5c), although the arrangement of the ROS was well preserved in the quercetin (+) retinas (Figure 5e).

### 3.5. Hsp70 Expression

**Figure 6:**
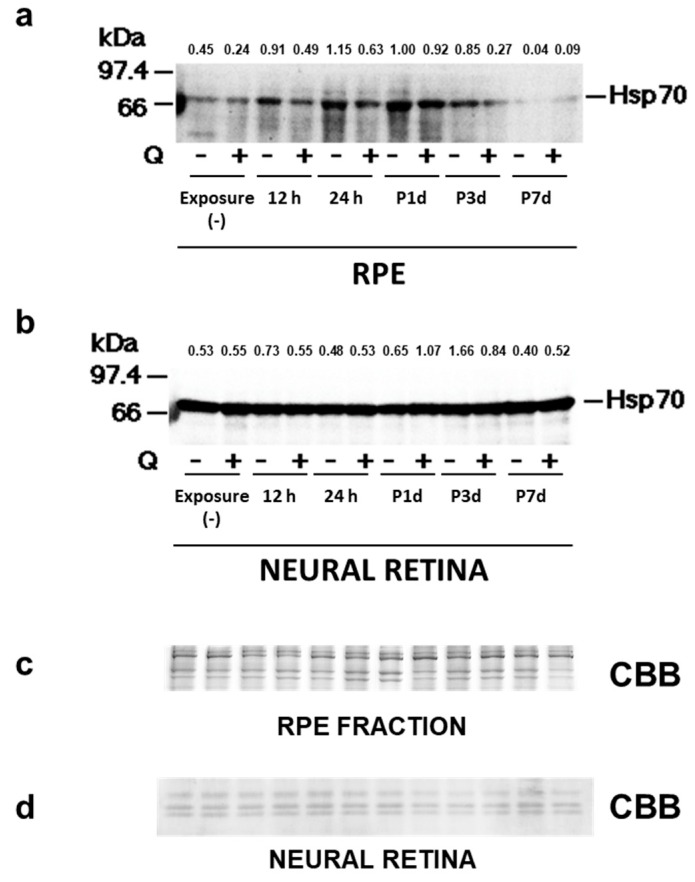
Expression of Heat shock protein 70 (Hsp70). Hsp70-specific labeling of about 66 kDa is seen and enhanced by light exposure. (**a**) The enhanced labeling is suppressed in quercetin (+) RPE cells. (**b**) Hsp70 labeling in the neural retina is unchanged by light exposure, and quercetin treatment does not affect the Hsp70 expression. The sample loading was monitored by staining with Coomassie Brilliant Blue (CBB) R-250, and the correction value with CBB is shown in the upper part. (**c**) RPE fraction and (**d**) neural retina. h, hours; d, day(s); and P, post.

Since quercetin is a potent inhibitor of Hsp70 and c-Fos [21,32], we evaluated the expression of those proteins in the retinal samples. In the quercetin (−) group, Hsp70 expression in the RPE fraction was up-regulated after 12 h of light exposure, peaked 1 day after light exposure, and subsided 7 days after light exposure (Figure 6a). In the quercetin (+) group, Hsp70 expression in the RPE fraction was lower than in the quercetin (−) group at all time points analyzed (Figure 6a). In the neural retina, Hsp70 expression was also up-regulated 1 or 3 days after light exposure in both groups and was lower in the quercetin (−) group (Figure 6b).

### 3.6. AP-1 Transcriptional Activity in Neural Retina

**Figure 7:**
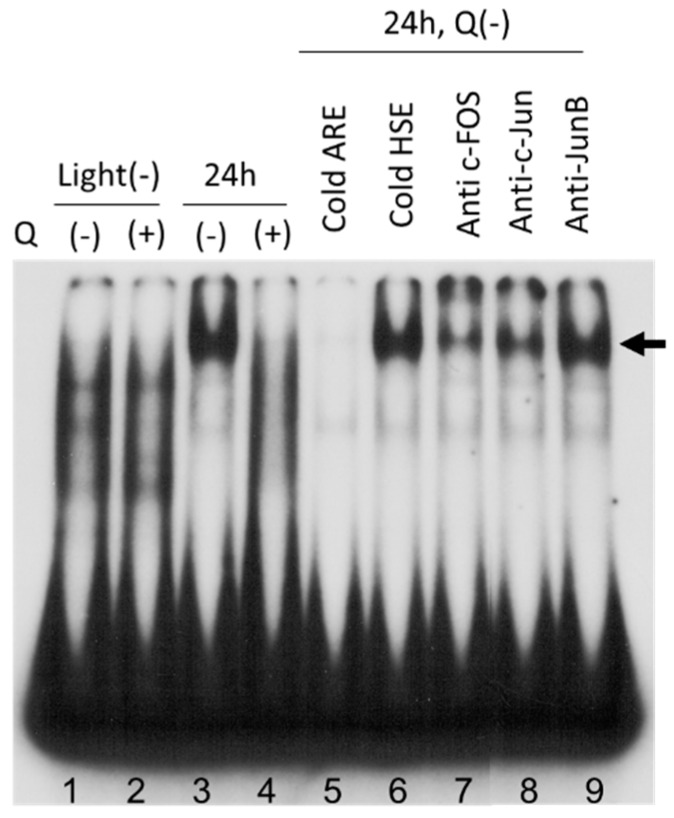
The electrophoretic mobility shift assay (EMSA). Nuclear proteins in the neural retinas from quercetin (+) or saline-treated rats are probed with a radio-labeled AP-1 sequence. Samples from rats not exposed to light are seen in lanes 1 and 2, and samples exposed to light for 24 h are seen in lanes 3–9. The rats were treated with saline (lanes 1, 3, and 5–9) or quercetin (lanes 2, 4). For specificity analysis of detected bands (arrow) of the AP-1-protein binding complex, the samples were preincubated with 100-fold molar excess of cold AP-1 probe (lane 5) or cold heat shock element probe (lane 6), before the radio-labeled AP-1 probe was added. The samples were preincubated with antibodies for c-Fos (lane 7), c-Jun (lane 8), and JunB (lane 9) before the radio-labeled AP-1 probe was added. Q, quercetin; h, hours; ARE: Antioxidant-responsive Element; HSE: Heat Shock Elements.

Since AP-1-mediated signal transduction plays a crucial role in photoreceptor cell apoptosis induced by light exposure [4], we evaluated the involvement of AP-1 in quercetin-mediated cytoprotection against retinal light damage. In the EMSA (Figure 7), no band was detected before light exposure in either the control (lane 1) or quercetin (+) rats (lane 2). With 24-h light exposure, the band was augmented markedly in quercetin (−) rats (lane 3), but not in quercetin (+) rats (lane 4). The band was offset completely by the cold AP-1 probe (lane 5) but not by the cold heat shock element (lane 6), suggesting that this band was specific for AP-1. The band was abrogated with the addition of antibodies against c-Fos (lane 7) and c-Jun (lane 8), but not by an antibody against JunB (lane 9).

## 4. Discussion

The ERG amplitudes were significantly higher in the quercetin (+) group than in the quercetin (-) group (Figure 1). Significantly more rows of photoreceptor cell nuclei were present in quercetin (+) rats compared to quercetin (−) rats 1 day after light exposure and thereafter (Figure 2m). Expression of TUNEL-positive cells after light exposure was inhibited markedly in quercetin (+) rats compared to quercetin (−) rats at all time points analyzed after light exposure (Figure 3). The ultrastructure of the ROS, which was the primary site of light-induced retinal damage, was well preserved morphologically in quercetin (+) rats but not in quercetin untreated (−) rats. These results indicated that pretreatment with quercetin inhibits light-induced photoreceptor cell apoptosis and subsequent retinal degeneration in rats.

In quercetin (−) group, it appears that retinas were normalized by day 7 without the need for quercetin (Figure 3, Figure 4, Figure 5 and Figure 6). In this animal model, oxidative stress induced by light exposure is transient, not continuous [31,32,33]. While retinal damage was detected by TUNEL or 8-OHdG immune-staining after exposure, those changes resolved. However, retinal damage induced by oxidative stress was not recover, and retina might beome thin.

Retinal tissues, particularly the ROS, contain high levels of polyunsaturated fatty acids compared to other tissues and might be affected easily by oxidative insult [35]. Shedding of the ROS from the photoreceptor cells and phagocytosis of the shed ROS by RPE cells are accelerated by light exposure [36]. Phagocytosed ROS are seen as phagosomes in the cell bodies of the RPE cells; thus, the amount of phagosomes in the RPE cells reflects the severity of the photooxidative stress in the photoreceptor cells. The rat RPE begins to phagocytize the ROS immediately after the start of light exposure, and the peak of phagocytosis is 2 h later [29,30]. In this study, we started light exposure and enucleation at 9:00 AM, which was 2 h after the light exposure started, in all groups except for the group exposed to light for 12 h. Therefore, the changes in the phagosomes was affected by light exposure, not circadian rhythm. After light exposure, there were significantly more phagosomes in the RPE cells in the quercetin (−) group than in the quercetin (+) group at all time points analyzed (Figure 5f). This suggested that the level of tissue oxidative stress is lower in the latter group than in the former group. Actually, light-induced up-regulation of 8-OHdG, an established marker of DNA oxidation, in the RPE and ONL was suppressed markedly by quercetin treatment. These results indicated that quercetin is an effective antioxidant against light-induced retinal damage in vivo. Previously, the cytoprotective effects of antioxidants such as ascorbate [36], dimethylthiourea [37], N-acetylcysteine [38], and phenyl-N-tert-butylnitrone (PBN) [39] have been reported. Our results further emphasized the role of antioxidants against retinal photic injury.

Hsp70 includes two functionally distinct isoforms, the constitutive form, Hsc70, and the stress-inducible form, Hsp70. In the retina, Hsp70 expression increases with retinal ischemia-reperfusion and intense light exposure [40,41]. Induction of Hsp72 in the neural retina by hyperthermia [42] and intravitreous injection of Hsp72 protein [43] contribute to retinal protection against light damage. The expression of c-Fos protein was up-regulated transiently by light exposure [7,9,10]. Lack of c-Fos prevents light-induced apoptosis in the photoreceptors [9,10]; thus, AP-1, a Jun/Fos heterodimeric transcription factor-mediated signal transduction was required for the intense-light-induced photoreceptor cell apoptosis [44]. Heat shock pretreatment up-regulates c-Fos and Hsp70 expressions in primary cultured astrocytes and pretreatment with quercetin inhibits the expression [21]; thus, Hsp70 and c-Fos seem to have a common upstream pathway for their induction. Therefore, the current study focused on Hsp70 and AP-1 to analyze further the cytoprotective mechanism of quercetin. In this study, Hsp70 expression was up-regulated markedly by light exposure in the quercetin (−) group (Figure 6a), suggesting that Hsp70 might be involved in the protection against retinal photooxidative stress, as indicated previously [34]. 

To confirm the contribution of the AP-1-mediated signal pathway to the effect of quercetin, we analyzed the DNA binding activity of AP-1 in light-exposed retina. In the EMSA, AP-1 DNA binding activity was activated by light exposure and binding of c-Fos and c-Jun, but JunB did not mediate binding activity (Figure 7), which agreed well with previous studies [45,46]. In quercetin-pretreated rats, light-mediated up-regulation of AP-1 activity was inhibited clearly (Figure 7). It is unclear if suppression of AP-1 activity is a direct effect of quercetin or a consequence of reduced oxidative stress. However, Yan et al. reported that quercetin inhibits cardiac hypertrophy by enhancing PPAR-c expression and suppressing AP-1 (c-Fos, c-Jun) activity, and that quercetin directly suppresses AP-1 activity in vitro [46]. Therefore, quercetin may protect the retina not only by an antioxidant effect but also in part by the suppression of AP-1 activity.

Anthocyanidins extracted from black rice, which are flavonoids like quercetin, have bioactivities as antioxidants and also prevent light-induced retinal damage via involvement of the AP-1 pathway [47]. Tomita et al. reported that down-regulation of AP-1 activity was involved in the radical trapping agent PBN-mediated cytoprotection against retinal light damage [39]. Taken together with the previous study, the current results suggested strongly that suppression of AP-1 binding activity is the common pathway for the effect of antioxidant properties against retinal photooxidative stress. The current study showed that quercetin protects against retinal damage induced by intense light. Although it is unknown if intraperitoneally injected quercetin reaches the retina, intraperitoneally injected PBN protects the retina from light-induced retinal damage in rats [39]. Dong et al. also administered quercetin intraperitoneally and confirmed that quercetin protected against oxidative stress and brain edema in an animal model of subarachnoid hemorrhage [27]. It also has been reported that oral administration of another flavonoid (green tea extract) reached the retina [48]. Therefore, it is possible that intraperitoneally injected quercetin was absorbed via the mesentery and reached the retina. Quercetin was reported recently to reduce apoptosis in the ischemia-reperfusion injury rat model [23] and prevents retinal neurodegeneration and oxidative stress in diabetic rats [49]. In vitro studies have shown the potential of quercetin as a ROS scavenger [50,51]. Furthermore, quercetin inhibits vascular endothelial growth factor-induced cellular proliferation and migration, suggesting that quercetin inhibits choroidal and retinal angiogenesis [52]. While further studies are needed, quercetin has the potential to become a multifunctional agent for protecting the retina in various retinal diseases.

## 5. Conclusions

In summary, intraperitoneal administration of quercetin down-regulates photooxidative stress in retinal tissue and mediates cytoprotection against light-induced photoreceptor cellular damage in rats. Suppression of the heterodimeric combination of c-Jun and c-Fos proteins to the AP-1 binding site is involved intrinsically in the quercetin-mediated cytoprotection. This study is the first to demonstrate that quercetin suppresses light-induced retinal degeneration via the AP-1 pathway.
